# Cost of illness of hepatocellular carcinoma in Japan: A time trend and future projections

**DOI:** 10.1371/journal.pone.0199188

**Published:** 2018-06-19

**Authors:** Kunichika Matsumoto, Yinghui Wu, Takefumi Kitazawa, Shigeru Fujita, Kanako Seto, Tomonori Hasegawa

**Affiliations:** 1 Department of Social Medicine, Toho University School of Medicine, Tokyo, Japan; 2 School of Nursing, Shanghai Jiao Tong University, Shanghai, China; Centre de Recherche en Cancerologie de Lyon, FRANCE

## Abstract

**Background:**

Hepatocellular carcinoma (HCC) is the fifth leading cause of death in Japan. The aim of this study was to calculate the social burden of HCC using the cost of illness (COI) method, and to identify the key factors driving changes in the economic burden of HCC.

**Methods:**

Utilizing government-based statistical nationwide data, the cost of illness (COI) method was used to estimate the COI for 1996, 1999, 2002, 2005, 2008, and 2014 to make predictions for 2017, 2020, 2023, 2026, and 2029. The COI comprised direct and indirect costs (morbidity and mortality costs) of HCC.

**Results:**

From 1996 to 2014, COI trended downward. In 2014, COI (579.2 billion JPY) was 0.71 times greater than that in 1996 (816.2 billion JPY). Mortality costs accounted for more than 70% of total COI and were a major contributing factor to the decrease in COI. It was predicted that COI would continue a downward trend until 2029, and that the rate of decline would be similar.

**Conclusions:**

COI of HCC has been decreasing since 1996. Treatment of patients infected with hepatitis C virus using newly introduced technologies has high therapeutic effectiveness, and will affect the future prevalence of HCC. These policies and technologies may accelerate the downward tendency of COI, and the relative economic burden of HCC is likely to continue to decrease.

## Introduction

Hepatocellular carcinoma (HCC; International Disease Classification 10 code C22) is the fifth leading cause of death (the fourth for males and the sixth for females) in Japan [1]. In many patients, HCC is caused by chronic hepatitis B virus (HBV) or hepatitis C virus (HCV) infection [2–4]. In Japan, it has been reported that 80% of HCC is caused by HBV or HCV [5]. HCC caused by chronic HCV infection is predominant in Japan, whereas HBV infection is the more predominant cause of HCC in other Asian countries [6, 7]. In recent years, the incidence of HCC caused by non-alcoholic steatohepatitis (NASH) has also been on the rise, and has become a social issue [2, 3].

Regarding the economic burden of HCC, several studies have reported the direct medical costs of HCC caused by HBV or HCV [8–13] or the social costs of HCC [14–19]. However, to date, few studies have attempted to estimate the total economic burden of HCC or other liver diseases in Japan [20, 21].

In this study, we calculated the cost of illness (COI), which included direct costs (DC) as well as indirect costs (IC) related to HCC, including opportunity cost resulting from disease and death. The objective of the study was to adequately capture the social burden of HCC by estimating past trends and projecting future trends in the cost of HCC.

We estimated the social burden of major forms of cancer from 1996 and projected the future social burden for major cancers [22–26]. These analyses showed that social aging and an increase in the average age of death had an impact on the decreased social burden by devaluing human capital. This study also focused on the relationship between social aging and trends in the COI of HCC.

## Methods

The COI method has been well described as a way to measure the social burden of disease [27–33]. In this study, COI was calculated from 1996 to 2014. Based on these data, future projections were made for the period from 2017 to 2029, to evaluate trends over time. The calculation method used in this study was the same as that used in our previous studies [20, 23].

COI is calculated as the sum of DC and IC, with IC divided into morbidity costs (MbC) and mortality costs (MtC), as per the following equation:

COI=DC+MbC+MtC

DC is the medical cost directly related to the disease (i.e., HCC), and include costs associated with treatment, hospitalization, testing, and drugs. In this study we calculated annual medical costs derived from the total medical expense data collected in the Survey of National Medical Care Insurance Services. DC of this survey comprised two parts: costs of inpatients (CI) and costs of outpatients (CO). These two costs are inclusive of drug costs. Therefore, DC can be defined as follows:

DC=CI+CO

MbC is the opportunity cost lost resulting from hospitalization and visits to hospital. We calculated MbC using the following equation:

MbC=TOVy×LVd/2+THD×LVd

where TOVy is the total person-days dedicated to outpatient visits, LVd is the one-day labor value per person, and THD is the total person-days of hospitalization. We calculated TOVy and THD in 5-year age groups based on the Patient Survey [34] conducted by the Japanese government every 3 years. We determined labor values by 5-year age group based on data from the Basic Survey on Wage Structure [35], the Labor Force Survey [36], the Estimates of Monetary Valuation of Unpaid Work, and the Evaluations of Domestic Labor [37]. We determined MbC by assuming a 1-day labor value loss per day in hospital and a half-day labor value loss per outpatient visit. We calculated LVd and THD as follows:

LVd=(Iy×ULVy)/365

THD=HP×ALOS

THD=HP×ALOS

where Iy is the annual income per person, ULVy is the annual monetary valuation of unpaid work per person, HPy is the annual number of hospitalized patients, and ALOS is the average length of hospital stay.

MtC is measured as the loss of human capital (human capital method), which was calculated using the following equation:

MtC=NDy×LVl

where NDy is the number of deaths and LVl is the lifetime labor value per person. We calculated LVl by summing the present value of the potential future income if the patient had survived. Regarding the potential future labor value, we conducted a sensitivity analysis of the discount rate. The base case discount rate was 2%, and our analyses included a discount rate varying between 0% and 5%.

Predictions of future COI from 2017 to 2029 were based on the Population Projection for Japan: 2016–2065 (January 2012) [38] conducted by the National Institute of Population and Social Security Research. We used a projection that assumed medium fertility and medium mortality. The year 2014 was selected as the benchmark for the 1-day labor value by sex and 5-year age group.

Two methods were utilized for the future projection of COI. The first was the “fixed” method (model 1), which fixed four health-related indicators (mortality rate, number of outpatient visits per population, number of hospitalizations per population, and average length of hospital stay) of each age group and the unit cost of CI and CO at the 2014 level and changed only future population and age structure. The other was the “variable” method, which estimated health-related indicators in addition to population and age structure. Future health-related indicators and the unit cost of CI and CO were estimated using linear regression (linear model; model 2); logarithmic regression for upward trend or exponential regression for downward trend regression (logarithmic/exponential model; model 3); or a combination of regressions of higher coefficient of determination (mixed model; model 4). We found that the mixed model was the most valid for the current study. We divided DC into hospitalization costs and outpatient costs, and calculated inpatient cost per day (iCd) and outpatient cost per day (oCd) by dividing by THD and TOVy, respectively. Future iCd and oCd were calculated by linear regression of past trends. We calculated each model’s DC by multiplying future iCd and oCd by the THD and TOVy values projected by each model.

To estimate and project COI values for HCC, we calculated the contribution ratio of DC, MbC, and MtC to the overall COI variation, as follows:

$$\frac{{Cost}_{t}^{i}-{Cost}_{0}^{i}}{{COI}_{t}-{COI}_{0}}$$

${Cost}_{t}^{i}$: DC, MbC, and MtC costs at year t;

${Cost}_{0}^{i}$: DC, MbC, and MtC costs at the baseline year;

*COI_t_*: COI at year t;

*COI*_0_: COI at the baseline year.

In addition, a sensitivity analysis was conducted in order to consider the effect of direct-acting antiviral agents (DAA), which were recently approved. Because the first DAA (telaprevir) was approved in 2011 in Japan, the effect of the previous trend of health-related indicators could not be estimated. Sofosbuvir (Harvoni®, Sovaldi®), which was approved in 2015, showed a highly sustained virological response. Therefore, we assumed that the effect of DAA on HCC caused by HCV will start from 2020, and we varied the annual decrease rate of health-related indicators (mortality rate, number of outpatient visits per population, and number of hospitalizations per population) between 0% (original analysis) and 20%. The mixed model was used. If we assume the annual decrease rate to be 20%, the health-related indicators will decrease to approximately 90% of those at 2017 in 2029. Therefore, we considered the maximum rate as 20%.

The protocol of this study was approved by the Ethical Committee of Toho University School of Medicine (reference number A16019).

## Results

Table 1 shows the trend of COI and health-related indicators from 1996 to 2014. COI was calculated to be 607.2 billion Japanese yen (JPY) in 2014. The contributions of DCs, MbCs, and MtCs to COI were 131.6 billion JPY, 18.5 billion JPY, and 457.1 billion JPY, respectively. MtCs were the greatest contributors and accounted for 75.3% of total COI. COI decreased continuously from 1996 to 2014 by 1.9% annually, representing a total 0.70-fold decrease. DCs increased until 2002 and decreased gradually thereafter. MbCs decreased from 1999, and MtCs which accounted for more than 70% of COI decreased starting in 2002. The contribution ratio of MtC to total decrease was 106.9%.

**Table 1:** The time trend of cost of illness (COI) of liver cancer.

	1996	1999	2002	2005	2008	2011	2014
Population (thousand persons)	125,864	126,686	127,435	127,768	127,692	127,799	126,949
[% of 65 years or older]	15.10%	16.70%	18.50%	20.20%	22.10%	23.30%	26.10%
Number of deaths (persons)	32,169	33,814	34,634	34,265	33,659	31,831	29,541
[% of 65 years or older]	63.1%	70.2%	75.5%	78.4%	81.3%	83.2%	86.9%
Incidence (persons)	40,128	39,816	40,604	42,194	48,512	43,840	43,667
[% of 65 years or older]	62.8%	67.9%	70.8%	72.1%	76.7%	78.7%	79.2%
Crude mortality/incidence rate	80.2%	84.9%	85.3%	81.2%	69.4%	72.6%	70.3%
Average age at incidence (years)	67.5	68.5	69.5	70.1	71.5	72.6	72.8
Average age at death (years)	67.9	69	70.5	71.9	73.2	74.5	75.8
Direct cost (billion JPY)	103.7	122.2	156.2	147.6	140.7	138.4	131.6
Morbidity cost (billion JPY)	28.7	35	34	31.5	26.4	21.4	18.5
Mortality cost (billion JPY)	730.7	684.2	719	624.6	578.3	548.6	457.1
[% of 65 years or older]	27.0%	32.4%	42.1%	44.7%	48.1%	54.7%	58.5%
Mortality cost per person (million JPY)	22.7	20.2	20.8	18.2	17.2	17.2	15.5
COI (billion JPY)	863.1	841.5	909.2	803.8	745.4	708.4	607.2

Source of population:Ministry of Internal Affairs and Communications "Population Estimates"

Source of the number of cancer deaths: "Vital Statistics"

Source of the number of incidence: Center for Cancer Control and Information Services, National Cancer Center, Japan.

Average age at incidence: Calculated according to the number of incidence.

Average age at death: Calculated according to the number of deaths, sex and age (5-year-old age-grade), cause of death in “Vital Statistics.”

The data of incidence, Crude mortality/incidence rate and Average age at incidence are 2012 data.

JPY: Japanese yen.

Decreased MtCs were the primary factor contributing to decreased COI. MtC per person (MtC/number of deaths) decreased continuously (31.9% decline; 22.7 million JPY in 1996 to 15.5 million JPY in 2014); moreover, NDy decreased (8.2% decline; 32,169 deaths in 1996 to 29,541 deaths in 2014). According to the National Cancer Center of Japan, the incidence of HCC increased by 8.8%, from 40,128 in 1996 to 43,667 in 2012 [39]. It is considered that the decreased NDy was caused by the decreased fatality rate. The mortality rate to incidence rate ratio also decreased by 10% from 1996 to 2014. On the other hand, the decreased MtC per person was considered to be due to increased age at death. During this period, the proportion of deaths in individuals aged 65 years and older increased from 63.1% to 86.9%, and average age of death increased from 67.9 to 75.8 years. The increase in deaths among elderly individuals with low human capital value was considered to decrease MtC per person. Table 1 shows the comparison of mortality rate, number of deaths, and incidence rate by sex and 5-year age group between 1996 and 2014.

Table 2 shows the future projection of COI from 2014 to 2029. Future projections of COI were calculated based on four models: the fixed, linear, logarithmic/exponential, and mixed models. In the fixed model, COI was projected to increase slightly from 607.2 billion JPY in 2014 to 646.3 billion JPY in 2029. DCs increased continuously, and MbCs and MtCs increased until 2020 and were stable thereafter. The contribution ratio of MtC to total increase was 44.9%. The fixed model assumed that health-related indicators and the unit cost of CI and CO were fixed at 2014 levels and that only demographic changes had any impact on COI. NDy increased 25.6% and the average age of death also increased by 2.3 years due to social aging. This was reflected in the increase of MtC. This model did not consider any past trends of health-related indicators. We treated this model as a reference only.

**Table 2:** Future prediction of cost of illness (COI) of liver cancer.

	Item	2014	2017	2020	2023	2026	2029
	Estimated population (thousand persons)	126,949	125,739	124,100	122,122	119,891	117,465
	[% of 65 years or older]	26.1%	28.0%	29.1%	29.8%	30.5%	31.2%
Fixed model	Number of liver cancer deaths (persons)	29,541	31,531	33,187	34,768	35,859	37,089
	[% of 65 years or older]	86.9%	88.6%	89.3%	89.5%	89.6%	89.6%
	Average age at death (years)	75.8	76.4	76.8	77.3	77.6	78.0
	Direct cost (billion JPY)	131.6	137.6	143.5	148.0	150.9	152.2
	Morbidity cost (billion JPY)	18.5	18.8	19.2	19.4	19.4	19.4
	Mortality cost (billion JPY)	457.1	463.4	469.7	473.1	475.3	474.6
	[% of 65 years or older]	58.5%	61.0%	61.5%	61.2%	60.8%	60.3%
	Mortality cost per person (million JPY)	15.5	14.7	14.2	13.6	13.3	12.8
	COI (billion JPY)	607.2	619.8	632.4	640.5	645.7	646.3
Linear model	Number of liver cancer deaths (persons)	29,541	30,192	29,844	30,891	31,924	32,732
	[% of 65 years or older]	86.9%	94.0%	96.0%	96.9%	97.1%	97.0%
	Average age at death (year)	75.8	78.1	79.5	80.3	80.7	81.5
	Direct cost (billion JPY)	131.6	137.6	131.6	128.2	122.3	126.3
	Morbidity cost (billion JPY)	18.5	17.1	14.4	12.2	10.3	9.4
	Mortality cost (billion JPY)	457.1	354.7	292.5	271.6	263.7	251.1
	[% of 65 years or older]	58.5%	73.3%	79.5%	83.2%	83.5%	82.5%
	Mortality cost per person (million JPY)	15.5	11.7	9.8	8.8	8.3	7.7
	COI (billion JPY)	607.2	509.3	438.5	412.1	396.3	386.7
Logarithmic / exponential model	Number of liver cancer deaths (persons)	29,541	32,357	32,690	33,238	33,686	33,926
	[% of 65 years or older]	86.9%	90.6%	92.3%	93.6%	94.6%	95.4%
	Average age at death (year)	75.8	76.9	77.8	78.7	79.4	80.2
	Direct cost (billion JPY)	131.6	141.9	142.2	140.8	137.6	132.0
	Morbidity cost (billion JPY)	18.5	18.9	17.3	15.6	13.9	12.3
	Mortality cost (billion JPY)	457.1	443.0	405.2	372.2	345.9	316.7
	[% of 65 years or older]	58.5%	65.0%	68.2%	70.7%	73.3%	75.6%
	Mortality cost per person (million JPY)	15.5	13.7	12.4	11.2	10.3	9.3
	COI (billion JPY)	607.2	603.8	564.7	528.5	497.4	461.0
Mixed model	Number of liver cancer deaths (persons)	29,541	31,954	31,943	32,504	33,196	33,721
	[% of 65 years or older]	86.9%	91.3%	93.7%	95.4%	96.2%	96.6%
	Average age at death (year)	75.8	77.2	78.4	79.4	80.0	80.8
	Direct cost (billion JPY)	131.6	142.9	141.7	140.6	140.0	142.8
	Morbidity cost (billion JPY)	18.5	18.4	16.2	14.2	12.8	11.8
	Mortality cost (billion JPY)	457.1	417.5	359.4	321.9	301.7	280.3
	[% of 65 years or older]	58.5%	66.8%	72.7%	77.5%	79.8%	80.8%
	Mortality cost per person (million JPY)	15.5	13.1	11.3	9.9	9.1	8.3
	COI (billion JPY)	607.2	578.8	517.3	476.7	454.5	434.9

JPY: Japanese yen.

On the other hand, in the variable models (linear, logarithmic/exponential, and mixed models) the trends were different from that of the fixed model. Firstly, the linear model showed that COI decreased by 36.3% from 607.2 billion JPY in 2014 to 386.7 billion JPY in 2029. DCs were stable, whereas MbCs and MtCs decreased continuously. NDy increased by 10.8% and average age of death increased by 5.7 years. The contribution ratio of MtC to total decrease was 93.5%.

In the logarithmic/exponential model, COI decreased by 24.1% from 607.2 billion JPY in 2014 to 461.0 billion JPY in 2029. DCs were stable, MbCs increased until 2017 and decreased thereafter, and MtCs decreased continuously. NDy increased by 14.8% and average age of death increased by 4.5 years. The contribution ratio of the mortality cost to total decrease was 96.0%.

**Fig 1:**
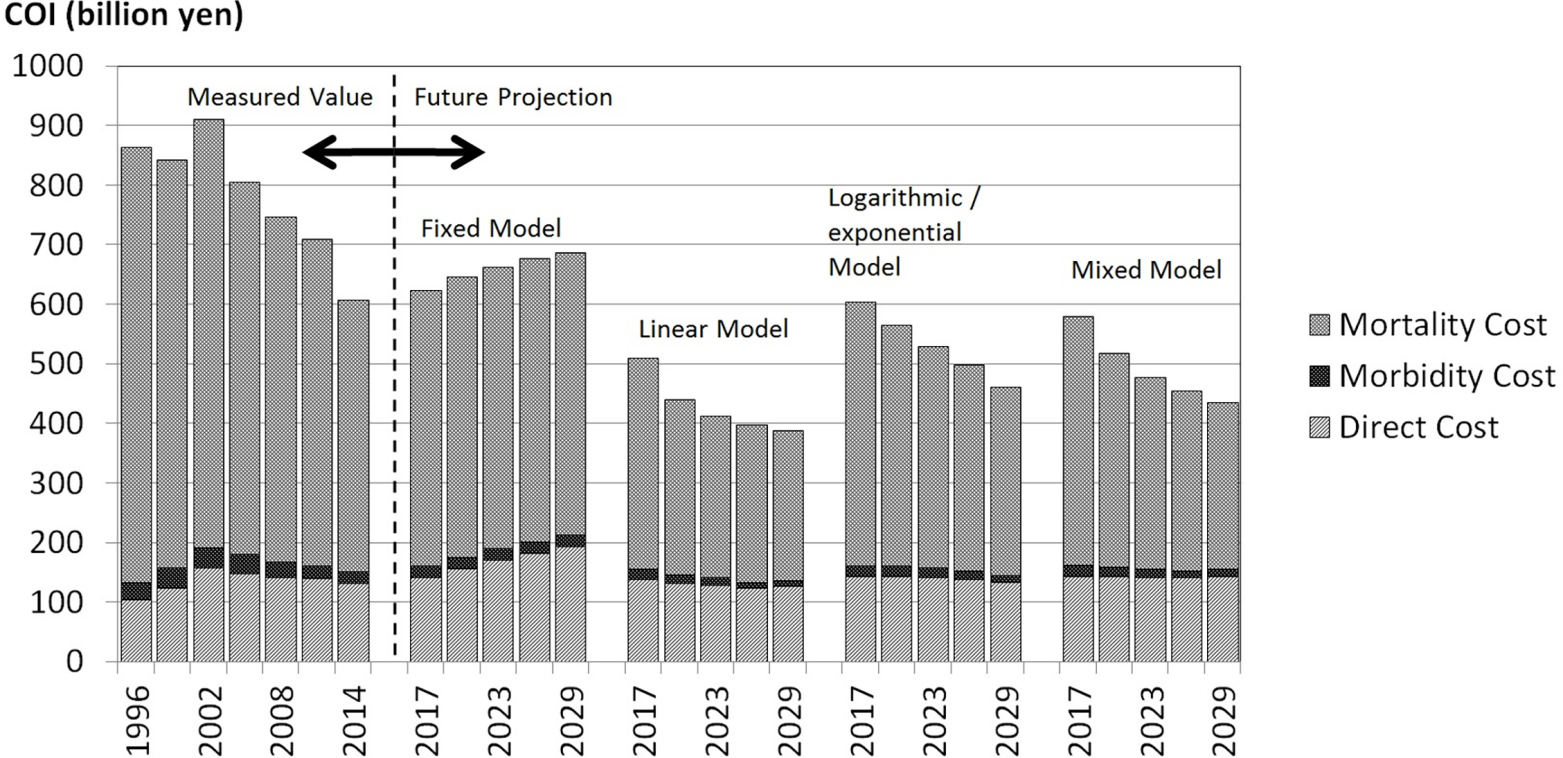
Cost of illness (COI) projection with cost element.

The mixed model was a combination of models of higher coefficient of determination and was therefore considered the most valid model used in this study. This mixed model showed that COI decreased by 28.4% from 607.2 billion JPY in 2014 to 461.0 billion JPY in 2029. DCs were stable, whereas MbCs and MtCs decreased continuously. NDy increased by 14.2% and average age of death increased by 5.1 years. The contribution ratio of MtC to total decrease was 102.6%. Fig 1 shows the trends of the individual COI components in each model.

Results of the sensitivity analysis for the mixed model are shown in Table 3. Estimations that assumed a 0% discount rate were 1.17–1.33 times higher than those that assumed a 5% discount rate, whereas trends of estimation were similar.

**Table 3:** Sensitivity analysis of discount rate: Mixed model.

				(billion Japanese yen)		
Year	Discount Rate					
	5%	4%	3%	2%	1%	0%
1996	738.7	775.0	816.2	863.1	917.1	979.8
1999	727.8	761.0	798.6	841.5	890.6	947.6
2002	787.0	822.7	863.1	909.2	962.1	1,023.4
2005	699.9	730.4	764.7	803.8	848.6	900.2
2008	649.5	677.6	709.3	745.4	786.9	834.8
2011	617.4	644.1	674.2	708.4	747.5	792.8
2014	532.7	554.5	579.2	607.2	639.3	676.5
2017	514.1	533.2	554.6	578.8	606.4	638.2
2020	464.6	480.2	497.7	517.3	539.7	565.3
2023	431.6	445.0	459.9	476.7	495.6	517.2
2026	413.9	426.0	439.5	454.5	471.4	490.6
2029	398.5	409.4	421.4	434.9	449.9	467.0

**Fig 2:**
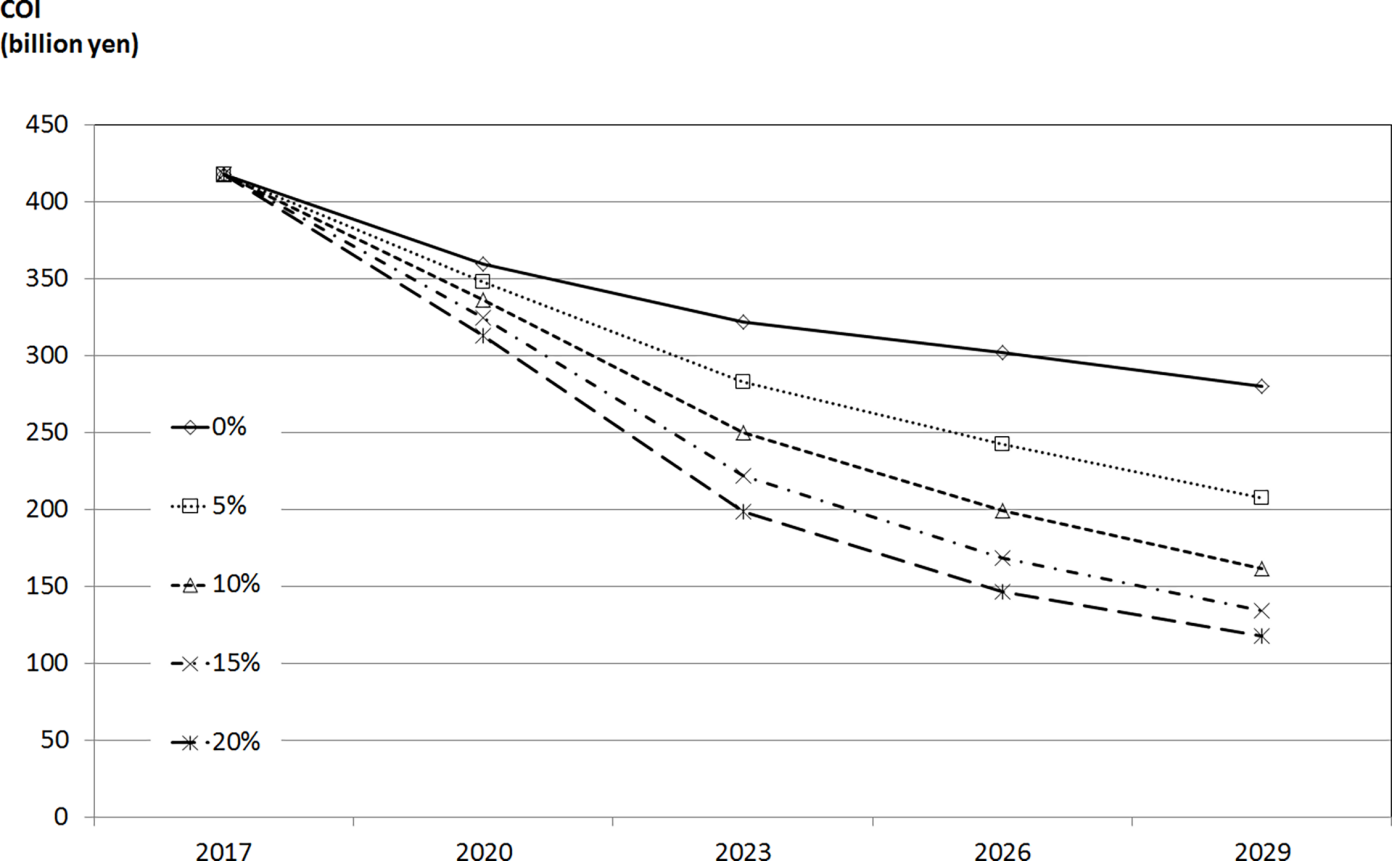
Sensitivity analysis of health-related indicators considering the decrease due to the effect of DAA.

Finally, the sensitivity analysis of health-related indicators considering the decrease due to the effect of DAA is shown in Fig 2. The COI at 2029 with 20% decrease rate was 0.420 times that in the original analysis (decreased rate due to DAA is 0%).

## Discussion

The results of this study demonstrated that after peaking in 2002, COI of HCC decreased. This decrease was attributed to decreased MtC. Furthermore, all variable models used herein predicted that COI would continue to trend downward until 2029. The mixed model showed that COI decreased continuously by 2.2% annually from 2014 to 2029, and this pace of decline was similar to that of the past (1.9% annually). The contribution ratio of MtC to total COI decrease was 106.9% in the past trend estimation, and was more than 90% in all three variable models. In fact, the MtC trend was considered to account for the majority of the COI trend.

Since MtC was calculated by multiplying NDy by LVl, the decreases in NDy and LVl were directly related to the decrease in MtC. LVl decreased continuously from 1996 and NDy of HCC decreased after peaking in 2002. LVl was influenced by a change in the annual labor value per capita and average age of death. Since the Japanese economy was in a severe condition, growth rate of the labor value per capita from 1996 to 2014 was 6.5%–that is, 0.3% annually. This had a small effect on the change of LVl during this period. However, increased average age of death during that period was remarkable (67.9 years old in 1996 to 75.8 years old in 2014), and impacted the decrease in LVl. LVl–that is, human capital value–differs by age, decreasing as one grows older. The increase in average age of death during that period thus led to a remarkable decrease in LVl.

On the other hand, NDy is influenced by social aging and changes in mortality rate in each age group. Because the mortality rate of HCC is higher in older age groups, the increased size of the elderly population due to social aging leads to an increase in NDy. The Japanese aging rate (percentage of population aged 65 years and older) increased from 15.1% in 1996 to 26.1% in 2014, and was considered to put upward pressure on NDy. In contrast, the decreased mortality rate in each age group was considered to have the opposite effect. According to Vital Statistics^1^, the age-adjusted mortality rate of HCC, for which the effect of age was removed, declined from 30.8 per 100,000 people in 1996 to 15.0 per 100,000 people in 2014. Considering these two effects, we determined that NDy of HCC increased until 2002 due to the social aging effect, but decreased thereafter because mortality rate effect exceeded the social aging effect.

We used such past downward trend of NDy of HCC. However, we believe that the future pace of decline of NDy will increase more rapidly than that forecasted by our projection. In Japan, the incidence of HCC caused by HBV and HCV, accounting for 80% of patients with HCC, has decreased in the long term [40–42]. Regarding HBV, in 1986 the Japanese government initiated a nationwide hepatitis B screening and immunization program for infants born to HBV carrier mothers. The number of HBV carriers since the 1986 birth cohort has decreased remarkably. Anti-HCV screening has also been performed since 1989, and use of nucleic acid technology to screen for HCV RNA was initiated in 1999. Additionally, several DAAs have been consecutively approved [43]. In our study, the effect of DAA on the incidence of HCC was determined using sensitivity analysis. The results showed that the COI decreased by 58% compared with that of the original analysis, considering 20% annual decrease rate of health-related indicators. However, the magnitude of the effect of DAA on the incidence of HCC is still controversial. Nevertheless, the future COI of HCC is expected to decrease more than that estimated by original projections.

Several previous studies in countries where HCV-related HCC was dominant showed that the direct cost of HCC was predicted to increase in the near future [10, 12, 13]. Regarding the effect of DAA, Sievert et al., Gane et al., and Duberg et al. showed the projection using their own model [11, 44, 45]. These three studies concluded that the incidence of or direct cost of HCV-related HCC would increase until approximately 2030, and accessibility to DAA is thus essential for reducing the incidence of or direct cost of HCC. In our estimation, direct cost of HCC already showed a downward trend since 2005 and was predicted to be stable until 2029 in mixed model. In Japan, selective vaccination targeting babies whose mothers were HCV carriers had contributed to a remarkable decrease in the number of HBV carriers since the 1986 birth cohort, and the introduction of more sensitive HCV antibody tests for blood transfusion in 1992 caused a remarkable decrease in HCV infections. In addition to these preventive methods, the Japanese public medical insurance covers treatments for HBV and HCV, including the second DAA, resulting in good access to the state-of-art technology. Previous studies have indicated that the accessibility to treatment in addition to the development of treatment is quite important for a substantial change in health-related indicators [11, 44–46]. This good accessibility is considered to be one of the factors contributing to the decrease in direct cost of HCC. Future studies should be conducted to clarify the effect of DAA on the incidence of HCC with its time frame in Japan, European countries, and USA, where HCV is the major cause of HCC. However, good accessibility to DAA is expected to accelerate the decline of the social burden of HCC in Japan.

Our study was not without limitations. Firstly, regarding the projection of DC, our projection only used hospitalization cost and outpatient cost of HCC to estimate DC of HCC. However, the treatment costs of HBV and HCV should also have been considered, because most cases of HCC result from HBV and HCV. In particular, recently developed direct-acting antiviral agents are highly effective and costly, and this study could not estimate the effect of these costs. These costs should be included in future research that aims to measure COI of comprehensive liver diseases including HCC, liver cirrhosis, and hepatitis. Additionally, the study period examined herein was relatively short and dramatic changes within the healthcare system occurred during this time. However, the variation among variable models was fairly low and as a result, our projections which predicted a decrease in COI–are likely to be accurate for the near future.

In the long term, COI of HCC is expected to decline, and both health policy and technological developments are expected to reduce the social burden of HCC even further. HCC can therefore be considered a successful case for health policy and technological development.

## Conclusion

The findings of the present study suggest that COI of HCC has decreased continuously to date and that this trend is likely to continue at a similar pace. During the study period, average age at death from HCC was older than that from other cancers and a rapid pace of aging was observed. These factors contribute to the decreased social burden of HCC. Moreover, past health policies and technological developments are considered to have accelerated the decrease in COI. We conclude that control policies for HCC are functioning effectively.
